# RELATIONSHIP BETWEEN SWALLOWING FUNCTION, NUTRITION, AND QUALITY OF LIFE DURING THE CORONAVIRUS DISEASE PANDEMIC

**DOI:** 10.1093/geroni/igae098.2492

**Published:** 2024-12-31

**Authors:** Naoko Morisaki, Keizo Numata

**Affiliations:** Himeji University, Himeji, Hyogo, Japan; Himeji Dokkyo University, Himeji, Hyogo, Japan

## Abstract

This study aimed to determine the relationship between swallowing function, nutrition, and quality of life (QOL) in older adults in Japan who stayed at home during this period of the COVID-19 pandemic. The participants of this study were adults aged 65 years or higher who were staying at home during the COVID-19 pandemic. A questionnaire-based survey was conducted from 2021 to 2022. The participants’ age, sex, swallowing function, nutrition, and QOL were surveyed. Swallowing function was assessed using the 12-item Dysphagia Risk Assessment for the Community-dwelling Elderly (DRACE) tool. Nutritional status was assessed using the Mini-Nutritional Assessment Short-Form (MNA-SF). QOL was assessed using the Short Form-8 (SF-8™) scale. Physical component summary (PCS) and mental component summary (MCS) scores were calculated based on the SF-8™ scale. Associations among swallowing function, nutrition, and QOL were analyzed using stepwise multiple regression analysis. The statistical software IBM SPSS Ver. 28.0 was used for the statistical analyses. The participants included 330 (70.1%) women and 141 (29.9%) men and their mean age was 81.5 ± 7.1 years. The participants’ average DRACE, MNA, PCS, and MCS scores were 3.65 ± 3.78, 10.25 ± 2.64, 46.31 ± 6.36, and 47.61 ± 6.31, respectively. The analysis was conducted with DRACE scores as the dependent variables and the age, sex, MNA, PCS, and MCS scores as the independent variables. A significant association was observed among swallowing function, nutritional status, and QOL (P < 0.01). The findings suggested an association among swallowing function, nutritional status, and QOL even during the pandemic.

